# Primary Isoniazid Mono-Resistant Pulmonary Tuberculosis in a COVID-19-Positive Male: World's First Case of Its Kind in the Present Pandemic

**DOI:** 10.7759/cureus.27163

**Published:** 2022-07-23

**Authors:** Sankalp Yadav

**Affiliations:** 1 Medicine, Shri Madan Lal Khurana Chest Clinic, New Delhi, IND

**Keywords:** rtpcr, rifampicin, isoniazid, covid-19, tuberculosis

## Abstract

The coronavirus disease 2019 (COVID-19) pandemic has resulted in numerous rare presentations of common diseases. The diseases that were prevalent for a long time like tuberculosis (TB) have been reported in cases of COVID-19. The reports of drug-sensitive, multidrug-resistant, and pre-extensively drug-resistant TB with COVID-19 are available in the medical literature. The situation in high TB burden countries where TB is a great contributor to morbidity and mortality is grisly. Herein, a case of primary isoniazid mono-resistant pulmonary tuberculosis in a COVID-19-positive Indian male is reported. As far as the literature is concerned, no case similar to our index case is ever reported.

## Introduction

Tuberculosis (TB) is a major public health problem and has been known for ages [1]. Nearly one-fourth of the world’s population is infected with *Mycobacterium tuberculosis* (MTB) [1]. Thereby making it one of the deadliest infectious diseases, with around 1.5 million deaths in 2020 [2]. As per the latest WHO statistics for the year 2021 from India, the incidence and prevalence of TB in India are 188 and 312 per one lakh (0.1 million) population [3]. This situation is notable as the National Tuberculosis Elimination Program (NTEP) is running as per the guidelines and efforts are aimed at TB elimination from India by 2025 [4]. The coronavirus disease 2019 (COVID-19) pandemic caused by severe acute respiratory syndrome coronavirus 2 (SARS-CoV-2) has ended up so far with extensive devastation in both the developed and the developing world [5]. The situation is alarming in countries where infectious diseases like TB have been prevalent [6]. This pandemic has to some extent negatively impacted national programs such as TB elimination programs [4].

Drug-resistant TB (DR-TB) constitutes a substantial part of all TB cases in high-burden countries [6]. The most prevalent form of DR-TB in the world is isoniazid-resistant, rifampicin-sensitive TB (HR-TB) that is resistant to drugs like isoniazid (H), which is a potent bactericidal drug and is an important part of TB management programs [7]. As per an estimate, HR-TB is seen in nearly seven percent of new TB cases and 8-11% of previously treated TB cases [8]. 

Reports of concomitant infections of COVID-19 and TB are sparse and are available in literature mainly as case reports [6]. Here, a case of primary isoniazid mono-resistant pulmonary tuberculosis in a COVID-19-positive Indian male is reported. A case of such presentation of HR-TB with COVID-19 has not yet been reported in the medical literature.

## Case presentation

A 20-year-old Indian male presented to our outpatient department (OPD) with chief complaints of cough with expectoration for two weeks, right-sided chest pain for one week, fever with night sweats for one week, and loss of appetite for one week with generalized weakness.

In the detailed history, the cough was continuous, associated with greenish-yellow colored non-foul smelling expectoration, and aggravated with exertion. Chest pain on the right side was mostly in the upper part of the chest and worsened when coughing. Fever mostly was low-grade, evening rise, and was relieved by taking over-the-counter antipyretics. He also mentioned the loss of appetite for a week and generalized weakness for around seven days.

This patient was a delivery agent by profession and was working during the COVID-19 pandemic except during local lockdowns. There was no history of substance abuse. His medical and surgical history was insignificant. Also, there was no history of any similar complaints in the past to him or to any of his family members. And there was no history of infectious diseases like TB or COVID-19 in the family or any contacts. Again, the patient was a non-immigrant with no history of imprisonment, unemployment, contacts with drug dealers, or commercial sex workers.

On general examination, he was afebrile with pulse of 101/minute, arterial blood pressure was 135/85 mmHg, respiratory rate was 30 breaths/minute, and SpO_2_ was 92% on room air. His SpO_2_ fell by 89% on room air after walking. Chest pain was aggravated on walking and subsided when he rested.

Systemic examination was remarkable with a dull note on percussion on the right hemithorax and vocal repercussions were diminished, and on auscultation, there was crepitation on the upper and middle lobes of the right lung. The rest of the systemic examination was unremarkable.

He was diagnosed as a probable case of TB and was given symptomatic treatment with cough syrup (ambroxol hydrochloride), and an antipyretic (paracetamol) besides, he was referred to the lab for his sputum for acid-fast bacilli (AFB) test, a cartridge-based nucleic acid amplification test (CBNAAT) of the sputum, a chest radiograph with other routine investigations including a reverse transcriptase polymerase chain reaction (RT-PCR) test for the SARS-CoV-2.

The results of sputum for AFB were suggestive of *Mycobacterium tuberculosis *(MTB) detected and the same was confirmed with CBNAAT. However, no resistance to rifampicin was detected. Per the NTEP guidelines, one sample was sent for line probe assay (LPA) and culture for drug susceptibility testing for first and second-line anti-tubercular drugs to the Intermediate Reference Laboratory (IRL).

**Figure 1:**
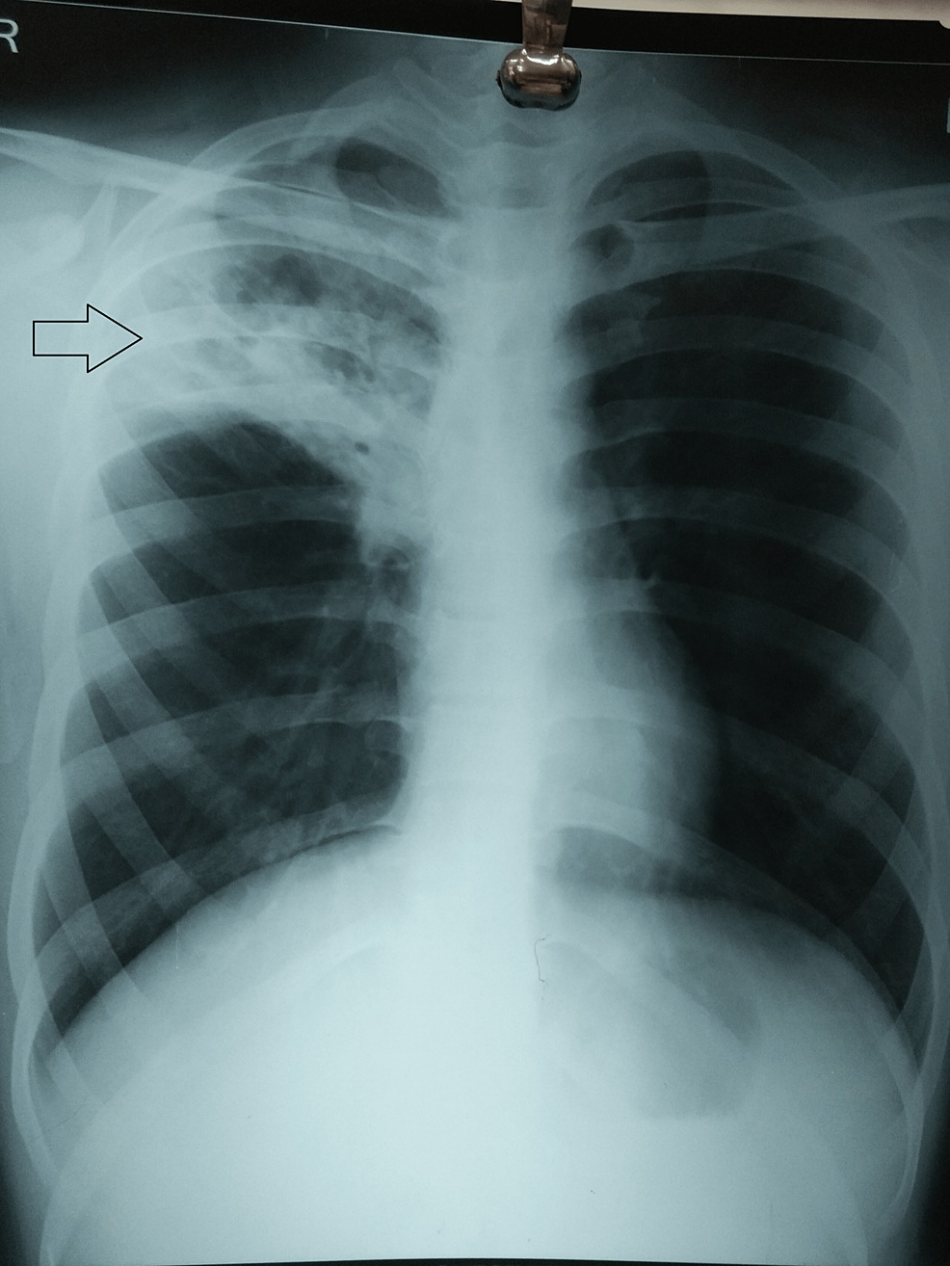
Chest radiograph posteroanterior (PA) view suggestive of consolidations at the upper lobe of the right lung with indistinct borders.

Meanwhile, RT-PCR was reported as positive for RNA specific to SARS-CoV-2 and the chest radiograph posteroanterior (PA) view was suggestive of consolidations at the upper lobe of the right lung with indistinct borders (Figure 1). Other investigations revealed a low lymphocyte count (1 × 10^7^/L), a high erythrocyte sedimentation rate (70 mm in the first hour), increased levels of C-reactive proteins (61 mg/L), and lactate dehydrogenase (LDH) (558 U/L). LPA results indicated resistance to isoniazid and sensitivity to rifampicin. Further, there was resistance to the katG gene.

Therefore, a diagnosis of primary isoniazid mono-resistant pulmonary tuberculosis with COVID-19 was made and treatment was initiated according to NTEP guidelines and national guidelines for COVID-19. He was started on rifampicin, ethambutol, pyrazinamide, and levofloxacin for six months as per his weight, and simultaneously symptomatic management for COVID-19 was started which included paracetamol, hydroxychloroquine, inhalational budesonide, vitamin B-complex, vitamin C, and betadine gargles. On his request, he was not admitted to the health facility and was kept under home isolation for two weeks with regular SpO_2_ monitoring and advised to report to the emergency department for any fall of oxygen saturation or any other major complaints. He was advised breathing exercises and yoga which helped him immensely and there were no episodes of fall of SpO_2_. After two weeks patient had a repeat RT-PCR from a private lab for COVID-19 and was found to be negative. Due to the patient's non-cooperation due to his weak economic status and the government's oversaturated free slots for CT scans, because of the pandemic, the same was not done. Thus, he was continued on the anti-TB regimen and regularly followed up in the OPD. Presently, the patient has completed five months of treatment and is doing well with no significant complaints. Upon his request to continue the remainder of his treatment, he was referred to his village. 

## Discussion

The present pandemic of COVID-19 has adversely affected the management of patients with other infectious diseases [6]. The reporting of TB cases was declining at the beginning of the pandemic [4]. As a result, there was a significant proportion of cases that either went unreported or died. The situation was the same, i.e., for both drug-sensitive and DR-TB cases [4].

DR-TB could be of many types, i.e., isoniazid (H)-resistant TB, rifampicin-resistant (RR)-TB and multi drug-resistant (MDR)-TB (RR and H resistant), pre-extensively drug-resistant TB (pre-XDR-TB) that is resistant to rifampicin (MDR/RR-TB) and any fluoroquinolone, and XDR-TB that is resistant to rifampicin (MDR/RR-TB), plus any fluoroquinolone, plus at the minimum to one of the drugs, bedaquiline and linezolid [4]. DR-TB is usually considered an outcome of improper treatment of drug-sensitive TB [9,10]. However, cases of primary drug resistance in the absence of any history of TB or any known contact are also available [6]. Concomitant infections of pulmonary TB with COVID-19 are rare and require a high index of suspicion as they share many similar clinical features [6]. In high TB burden countries, the pandemic has already overwhelmed healthcare systems and thus timely diagnosis backed with prompt treatment of both these infections is very important [6]. In a recent meta-analysis, the mean in-hospital fatality rates of cases with concomitant TB and COVID-19 were reported as 22.5% (95% CI: 19.0% to ~26.0%) in low/middle-income countries (India, Philippines, South Africa) [11]. This showed that there is a high fatality risk in the cases with both these infections [11]. However, there is a paucity of literature about prevalence, treatment, and long-term outcomes in these countries in COVID-19 with DR-TB cases.

Isoniazid is an important anti-TB agent because of its early bactericidal activity, low cost, and comparatively fewer side effects [1]. The development of H-mono-resistance is alarming as it is suggestive of negative treatment outcomes and progression to MDR-TB, especially in children and people living with HIV/AIDS [12]. Reports of primary H-mono resistant pulmonary TB in the absence of any history or any known contacts are even more alarming as such cases are extremely rare and a very high degree of suspicion is required to establish the diagnosis [6]. This case will serve as an important new addition to the existing knowledge about the management of HR-TB thereby helping the healthcare workers.

## Conclusions

The present case highlights the importance of a detailed history and clinical examination, especially in high TB settings. The signs and symptoms of several diseases resemble COVID-19 and thus it is imperative to diagnose the cases with an eye for rare or never seen before presentations of different diseases. This case would serve as an important addition to the literature as diagnosis of primary Isoniazid mono-resistance with COVID-19 is never reported to date and if untreated HR-TB is associated with an increased chance of developing further drug resistance and evolving towards MDR-TB.
